# Neural Correlates of Prosocial Behavior: Compensating Social Exclusion in a Four-Player Cyberball Game

**DOI:** 10.1371/journal.pone.0159045

**Published:** 2016-07-08

**Authors:** Mara van der Meulen, Marinus H. van IJzendoorn, Eveline A. Crone

**Affiliations:** 1 Institute of Psychology, Leiden University, Leiden, the Netherlands; 2 Center for Child and Family Studies, Leiden University, Leiden, the Netherlands; 3 Leiden Consortium Individual Development, Leiden University, Leiden, the Netherlands; University of Vienna, AUSTRIA

## Abstract

Prior studies demonstrated contributions of the insula and dorsal anterior cingulate cortex (dACC) for both experiencing and observing social exclusion, but it is not yet well understood how the brain processes the compensation of exclusion, as is observed in prosocial helping. Here, we tested if social brain regions, specifically the medial prefrontal cortex (mPFC) and temporal parietal junction (TPJ) are involved when individuals show prosocial behavior towards excluded others. For this purpose, 23 female participants played a four-player Cyberball Game in which participants could toss balls to each other. During the exclusion game, two players excluded one of the other players. When participants observed exclusion by others, they showed elevated activity in the insula, consistent with prior studies. However, when they tossed the ball to the excluded player, they showed increased activation in the TPJ, consistent with the hypothesis that prosocial behavior is associated with social reasoning. In addition, tossing to the excluded player was associated with increased activity in the nucleus accumbens (NAcc). Given that prior studies reported that the NAcc is involved in experiencing rewards, this may suggest a warm glow for showing prosocial compensation behavior when helping excluded others.

## Introduction

Prosocial behavior involves helping, sharing or comforting others without personal benefit, and is an important component of social life. Prior studies showed that humans compensate for others who are in need, for example by sharing distress of observed exclusion [1–3] and by helping victims of exclusion [4]. Prior studies also documented that seeing someone in distress causes feeling of personal distress as well [5]. Knowing you can do something to alleviate another person’s distress can lead to acts of prosocial behavior, as already seen in very young children [6]. Yet, prior studies have not attempted to separate the different components of sharing social pain and compensating others, possibly because it is difficult to disentangle these effects using behavioral measures. Neuroimaging may prove a powerful tool to dissociate which brain regions are sensitive to separate phases of observing social pain of others and actively compensating social exclusion.

Several earlier neuroimaging studies have shown the link between observed distress and experienced personal distress. These studies typically show that observing others in social pain activates similar brain regions as when being excluded [1, 7–9]. Prior studies have made use of a virtual ball tossing game referred to as Cyberball, in which three players participate in a computerized game of ball tossing. During the first round all players participate and toss the ball to each other. In subsequent rounds the computer controlled game ensures that one player no longer receives the ball, thereby creating a situation of social exclusion [10]. When individuals are excluded by others in Cyberball, they show increased neural activity in the dorsal anterior cingulate cortex (dACC) and bilateral insula [11–12]. Furthermore, there is consistent evidence for the role of dACC and bilateral insula in experiencing social rejection (for meta-analyses see [13–14]). The meta-analyses also revealed other areas implicated in social rejection, such as the left orbitofrontal cortex. However, the orbitofrontal cortex plays a more general role in human decision making and signaling value and might therefore be considered less specific to the experience of social rejection [15]. The observation of social exclusion has also been associated with activity in the bilateral insula and dACC, although more so when a friend (compared to a stranger) is being excluded [8]. Interestingly, several studies have reported that activity in the insula is correlated with subsequent prosocial behavior [1–2] and that activity in both bilateral insula and dACC is associated with empathy traits [2, 8].

A separate set of neuroimaging studies focused on the role of helping, or compensating for social pain of others. When participants are given the opportunity to interact with the players from a previously observed Cyberball game, participants tend to show more prosocial behavior towards the excluded player than towards the excluding players [1–3]. Prior studies tested how participants allocate points to others after playing Cyberball and being excluded. It was found that considering how to react towards excluders was associated with more activity in the temporal-parietal junction (TPJ; [16–17]), a region associated with perspective taking [18]. The TPJ is often interpreted as a brain region which is part of a social brain network, including also the medial PFC and superior temporal sulcus (STS), and which is activated when individuals think about intentions of others [19–20]. In addition, it was previously found that sharing with others results in increased activity in the ventral striatum/nucleus accumbens (NAcc), which is thought to be a reward center of the brain [21–24]. Possibly, prosocial behavior elicits a rewarding feeling, or a warm glow [25].

Taken together, prior studies showed that the insula and dACC are active when experiencing exclusion or observing exclusion of others [8, 11] and medial PFC, TPJ and NAcc are activated when compensating others or considering the reactions towards excluders [16–17, 22], but no study to date directly compared the neural correlates of observing exclusion and helping in a prosocial game. The goal of this study was to test the relative role of these regions in a prosocial Cyberball game in which participants can help excluded players. During this game, the participants observe another player being excluded, while having the opportunity to compensate for the excluding behavior by tossing the ball to the excluded player.

First, we predicted that participants would take the opportunity to compensate for observed exclusion by acting prosocially towards an excluded person [1–4]. We expected that this behavior would be related to self-reported empathy [2]. Second, we predicted that the observation of exclusion would lead to increased activity in the bilateral insula (replicating [1, 7]) and possibly dACC given its role in experiencing social exclusion [11]. Finally, we expected participants would show elevated responses in mPFC, TPJ and NAcc during acts of prosocial behavior (tossing the ball to the excluded player compared to tossing the ball to players who were not excluded) [17, 22].

## Materials and Methods

### Participants

The final sample consisted of 23 healthy female participants of 18 and 19 years old (*M* = 19.08, *SD* = .48). One additional participant was excluded due to a technical error when collecting the MRI data. Participants were recruited through local advertisements. All participants were screened for MRI contra indications and psychopathology using a telephone interview before the scanning session. This study was approved by the Commission Medical Ethics of the Leiden University Medical Center. Written informed consent was obtained from all participants prior to the scan session. Participants received €30 for participation in a larger set of studies.

### Experimental design

**Fig 1:**
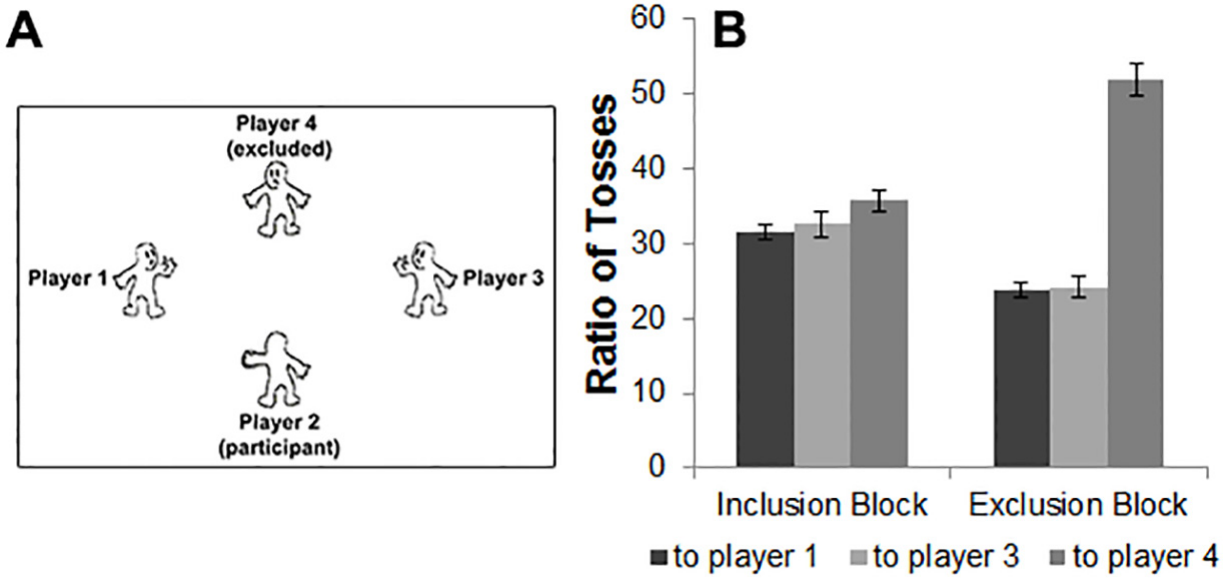
(A): Example of stimulus display (participant numbers are shown for illustration purposes only but were not presented to the participant), (B): Ratio of tosses from the participant to the other three players in the inclusion and exclusion block.

The Prosocial Cyberball game was an adapted version of the game used by Riem, Bakermans-Kranenburg, Huffmeijer and van IJzendoorn [4]. The participant was depicted by a classical Cyberball figure [10] at the bottom of the screen. The other three figures portraying the three other players in the game were positioned to the left and right of the screen center, and at the top of the screen (see Fig 1). Participants were told that they would play a computer game in which players toss balls to each other, and were asked to imagine that they were playing the game with other individuals. Various studies have shown that knowingly playing a game against a computer can also lead to feelings of exclusion. For example, no differences were found between conditions in which participants merely imagine that they are playing with others or believe that other players are really present [26]. Therefore, we used the manipulation of imagining playing with others. This is a powerful manipulation in research on gaming [27].

During the game, participants were instructed to toss the ball to the other players using a button box attached to their legs. The game consisted of two blocks. The first block was a fair situation, consisting of 120 trials. During this fair block all players received the ball an equal number of times. The second block was an exclusion situation, consisting of 168 trials. During this exclusion block, the player at the top of the screen (player 4) was excluded by the players positioned on the left and right of the screen center (players 1 and 3). Jitter was added at the end of each ball toss and ranged between 1000–2000 ms in steps of 500 ms.

Prosocial behavior during the game was measured by the ratio of tosses from the participant to the excluded player. This was calculated by dividing the number of tosses from the participant to the excluded player by the total number of tosses from the participant to any of the players. We expected to find a toss ratio of .33 from the participant for each player during the inclusion block, and a toss ratio larger than .33 from the participant to the excluded player during the exclusion block (based on [4]). A toss ratio larger than .33 (relative to tosses to the other players) would indicate compensation for the exclusion by the left and right player.

### Questionnaire

A Dutch version [28] of the Interpersonal Reactivity Index (IRI; [29] was used to assess empathy. The IRI is a widely used self-report measure of empathy with 4 subscales that assess perspective taking (e.g. “Before criticizing somebody, I try to imagine how I would feel if I were in their place”, 7 items, α = .73), empathic fantasy (e.g. “I really get involved with the feelings of the characters in a novel”, 7 items, α = .86), empathic concern (e.g. “I am often quite touched by things that I see happen”, 7 items, α = .86), and personal distress (e.g. “I sometimes feel helpless when I am in the middle of a very emotional situation”, 7 items, α = .63). Deleting one of the items (“I sometimes feel helpless when I am in the middle of a very emotional situation”) from the subscale personal distress yielded α = .69 after standardizing the remaining 6 items. All items can be answered on a five-point scale (0 = does not describe me well to 4 = describes me very well). On all subscales, high scores indicate higher levels of empathy.

### Procedure

Participants received explanations regarding the procedure of an fMRI scan and the Prosocial Cyberball game. After these explanations, participants performed five practice trials of the Prosocial Cyberball game. Directly after the scanning session participants were administered a pen-and-paper version of the IRI questionnaire.

### MRI data acquisition

Scans were made with a 3 Tesla Philips scanner, using a standard whole-head coil. The functional scans were acquired using a T2*-weighted echo-planar imaging (EPI). The first two volumes were discarded to allow for equilibration of T1 saturation effects (TR = 2.2 s, TE = 30 ms, sequential acquisition, 8 slices of 2.75 mm, field of view 220 mm, 80 × 80 matrix, in-plane resolution 2.75 mm). A high-resolution 3D T1-FFE scan for anatomical reference was obtained (TR = 9.760 ms; TE = 4.59 ms, flip angle = 8°, 140 slices, 0.875 × 0.875 × 1.2 mm3 voxels, FOV = 224 × 168 × 177 mm3). After the functional runs, a high resolution 3D T1-weighted anatomical image was collected (TR = 9.751 ms, TE = 4.59 ms, flip angle = 8°, 140 slices, 0.875 mm × 0.875 mm × 1.2 mm, and FOV = 224.000 × 168.000 × 177.333). Visual stimuli were shown on a screen that was attached in the magnet bore. Participants could see the stimuli via a mirror attached to the head coil. Head movement was restricted by using foam inserts inside the coil.

### fMRI data analysis

All data were analyzed with SPM8 [Wellcome Department of Cognitive Neurology, London]. Images were corrected for differences in rigid body motion. Structural and functional volumes were spatially normalized to T1 templates. Translational movement parameters never exceeded 1 voxel (3 mm) in any direction for any participant or scan. The normalization algorithm used a 12-parameter affine transform together with a nonlinear transformation involving cosine basis functions and resampled the volumes to 3 mm cubic voxels. Templates were based on the MNI305 stereotaxic space [30]. Functional volumes were spatially smoothed with an 6 mm FWHM isotropic Gaussian kernel.

The start of each ball toss was modeled separately using a zero duration event (see [17]). The modeled events were separated in three overall contexts, which distinguished between the participant a) observing the other players toss to each other (“Observed Exclusion”), b) tossing the ball (“Ball Tossing”), or c) receiving the ball (“Ball Receiving”). These conditions were separated to control for confounding factors such as motor actions or motor preparation. As can be seen in Fig 1A, the participant was referred to as player 2, the excluders were referred to as players 1 and 3, and the excluded player was referred to as player 4.

The “Observed Exclusion” context in which the participant observed others playing were separated in “Observed Excluding” (player 1 to player 3 or vice versa) and “Connecting” (excluded player 4 to other players 1 and 3). The “Ball Tossing” context in which the participant was tossing the ball was separated in “Compensating” (participant to excluded player 4) and “Tossing” (participant to player 1 and 3 combined). Finally, the “Ball Receiving” context in which the participant received the ball was separated in “From Excluder” (players 1 and 3 to participant) and “From Excluded” (excluded player 4 to participant). The condition in which the other players (1 and 3) tossed the ball to the excluded player (4) was modeled separately but was not further analyzed, because this event occurred only once in the exclusion game. Therefore three separate contexts with a total of six conditions were used in the analyses. All events were time-locked to the moment of the start of the ball toss. The trial functions were used as covariates in a general linear model; along with a basic set of cosine functions that high-pass filtered the data. The least-squares parameter estimates of height of the best-fitting canonical HRF for each condition were used in pair-wise contrasts. The resulting contrast images, computed on a subject-by-subject basis, were submitted to group analyses.

### Region of interest analyses

To test neural correlates of observed exclusion and prosocial behavior in the a priori defined brain regions, region-of-interest (ROI) analyses were performed with the Marsbar toolbox in SPM8 [31]. ROIs were selected based on previous studies showing associations with observing social exclusion (bilateral insula and dACC; [8]), compensating for social exclusion (mPFC and TPJ; [16–17]), and prosocial behavior (NAcc; [22, 25]). For dACC, a template based on a large scale reverse-inference analysis on social pain was used [32]. The website “Neurosynth” [http://www.neurosynth.org] was used to extract independent templates for bilateral insula, mPFC, and bilateral TPJ. A mask for bilateral insula was created with the search term ‘rejection’ (retrieved on April 30^th^, 2015), using forward inference with a standard threshold of Z > 2.3. Masks for mPFC and bilateral TPJ were created with the search term ‘mentalizing’ (retrieved on April 24^th^, 2015), using forward inference with a standard threshold of Z > 2.3. The resulting templates for bilateral insula and mPFC were then masked with anatomical masks for these areas because they were connected with other regions. An anatomical mask of the left and right NAcc was extracted from the Harvard-Oxford subcortical atlas, with the threshold at 40%. The center of mass for each ROI, as well as the volume of each ROI is presented in Table 1. Results from these ROIs were compared with repeated-measures ANOVAs for the three separate contexts: Observed Exclusion (Observed Excluding vs Connecting), Ball Tossing (Compensating vs Tossing), and Ball Receiving (From Excluders vs From Excluded). The context Ball Receiving is reported for completeness, but we had no a priori hypotheses about this condition.

**Table 1:** Coordinates and volumes of Regions of Interests that were extracted from Neurosynth and anatomical atlases (see text for details).

			Center of Mass		
Name	L/R	Volume (mm)	X	Y	Z
**dACC**		8880	1	15	30
**NAcc**	L	712	-10	12	-7
**NAcc**	R	696	10	13	-7
**Insula**	L	1352	-34	18	-1
**Insula**	R	1064	36	24	-1
**mPFC**		7304	-1	54	24
**TPJ**	L	7024	-50	-58	25
**TPJ**	R	7160	52	-55	23

### Whole brain analyses

To investigate neural responses on prosocial behavior across the brain we calculated the following contrasts, focusing on the exclusion game specifically. These analyses may reveal activity in brain regions other than the a priori selected areas. We tested the neural response to observing exclusion, with the contrast: Observed Excluding > Connecting (and the reversed contrast). We also tested the neural response to tossing prosocially to the excluded player, with the contrast: Compensating > Tossing (and the reversed contrast).

Task-related responses were considered significant if they exceeded a cluster-corrected threshold of *p* < .05 FDR-corrected, at an initial threshold of *p*< .005 [33].

## Results

### Behavioral results

To examine whether the participants compensated for the exclusion behavior by players 1 and 3 by tossing more balls to the excluded player (player 4), a repeated measures ANOVA with two within-subject factors was performed. Ratio of ball tosses from the participant to the other three players was the dependent variable (hereafter referred to as “player”), and play block (fair or exclusion) was the within-subject variable. In case the spherificity assumption was violated, we applied Greenhouse-Geisser corrections. The analysis resulted in a main effect of player (*F*(2, 44) = 35.02, *p* < .001, η_p_^2^ = 0.69), and a significant block*player interaction (*F*(2, 44) = 50.92, *p* < .001, η_p_^2^ = 0.77). No significant differences in ball tosses in the fair block were found (*F*(1, 22) = 1, *p* = .33). Post hoc contrasts revealed that in the exclusion block there was no significant differences in ratio of ball tosses to player 1 and 3 (*M* = 27.76, *SD* = .64, and *M* = 28.36, *SD* = 1.39, respectively). However, a significant difference was found in tosses to player 1 compared to player 4 (*F*(1, 22) = 52.64, *p* < .001, η_p_^2^ = 0.77), and between tosses to player 3 compared to player 4 (*F*(1, 22) = 78.16, *p* < .001, η_p_^2^ = 0.85; see Fig 1B). These findings show that participants compensated by tossing the ball more often to the excluded player.

In order to check whether behavior during the Prosocial Cyberball game was associated with self-reported empathy, we correlated the results of the IRI subscales with the ratio of ball tosses to the excluded player. There were no significant correlations with these subscales (see Table 2).

**Table 2:** Correlations between behavior during the exclusion game and self-reported empathy.

		*M* (*SD*)	PT	FS	EC	PD	To player 1	To player 3	To player 4
*IRI subscales*	PT	16.91 (4.17)							
	FS	19.73 (5.39)	.32						
	EC	19.96 (5.64)	.53*	.53**					
	PD	12.87 (4.13)	-.37	.23	.11				
*Ratio of tosses during PCG*	To player 1	23.90 (4.86)	.02	.14	.36	.29			
	To player 3	24.14 (6.78)	.19	-.06	.35	.16	.57**		
	To player 4	51.96 (10.35)	-.13	.03	-.40	-.24	-.84**	-.92**	

* *p* < .05

** *p* < .01, PT = Perspective Taking, FS = Fantasy Scale, EC = Empathic Concern, PD = Personal Distress, PCG = Prosocial Cyberball Game.

### ROI analysis

**Fig 2:**
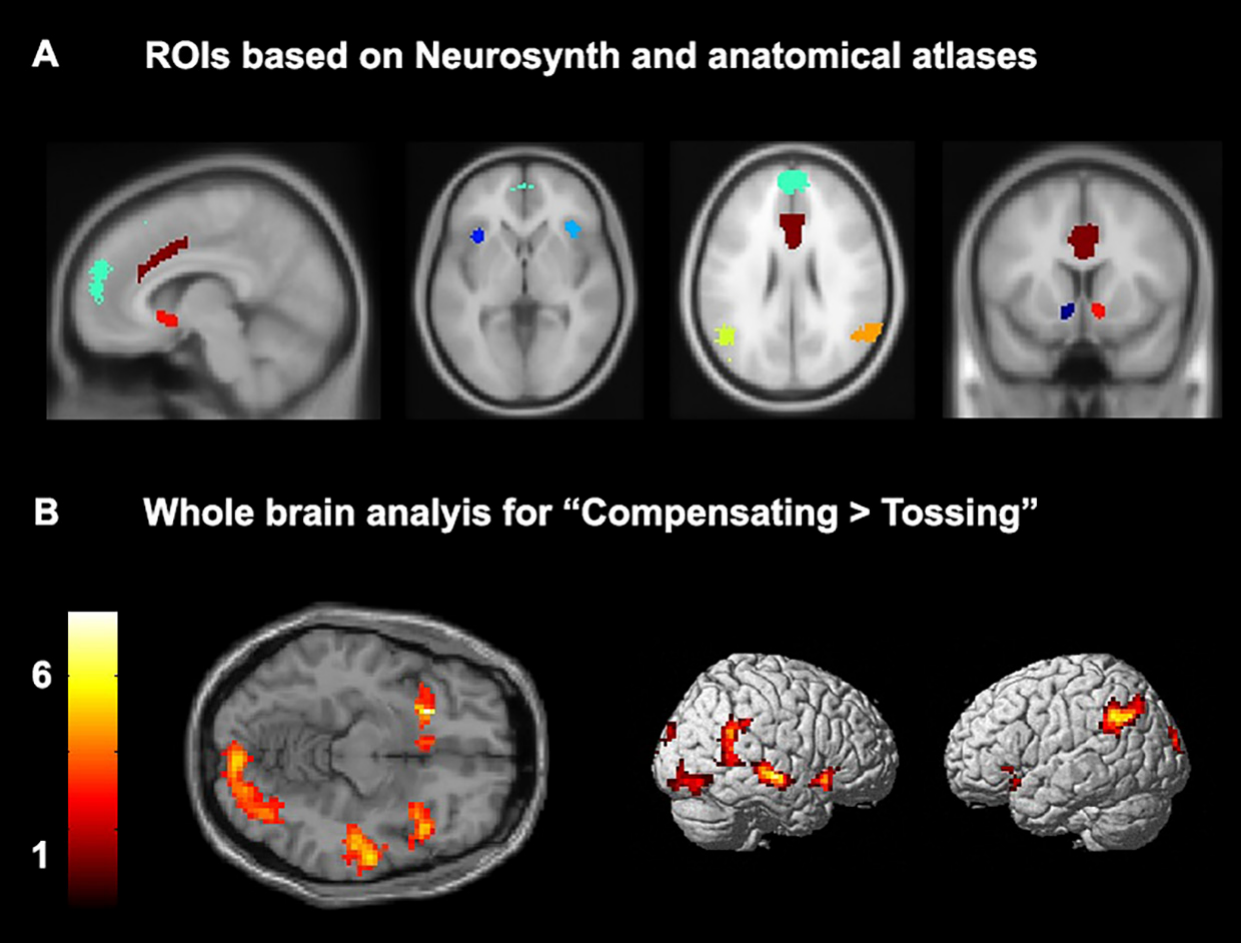
Representation of (A) Independently selected ROIs: dACC (dark red, most left picture), mPFC (turquoise, most left picture) bilateral insula (blue, second picture to the left), bilateral TPJ (green and orange, second picture to the right), and bilateral NAcc (dark blue and red, most right picture). See text for explanation of the selection procedure. See Table 1 for coordinates of center of mass. (B) Whole brain results for the contrast “Compensating > Tossing”, for a cluster-corrected threshold of *p* < .05 FDR-corrected, 101 contiguous voxels, at an initial threshold of *p* < .005.

We performed region of interest analyses for insula, dACC, mPFC, TPJ,and NAcc (Fig 2) for the three separate contexts: Observed Exclusion, Ball Tossing, and Ball Receiving. Repeated measures ANOVAs were performed for each region and for each of the three contexts separately.

#### Observed Exclusion

**Fig 3:**
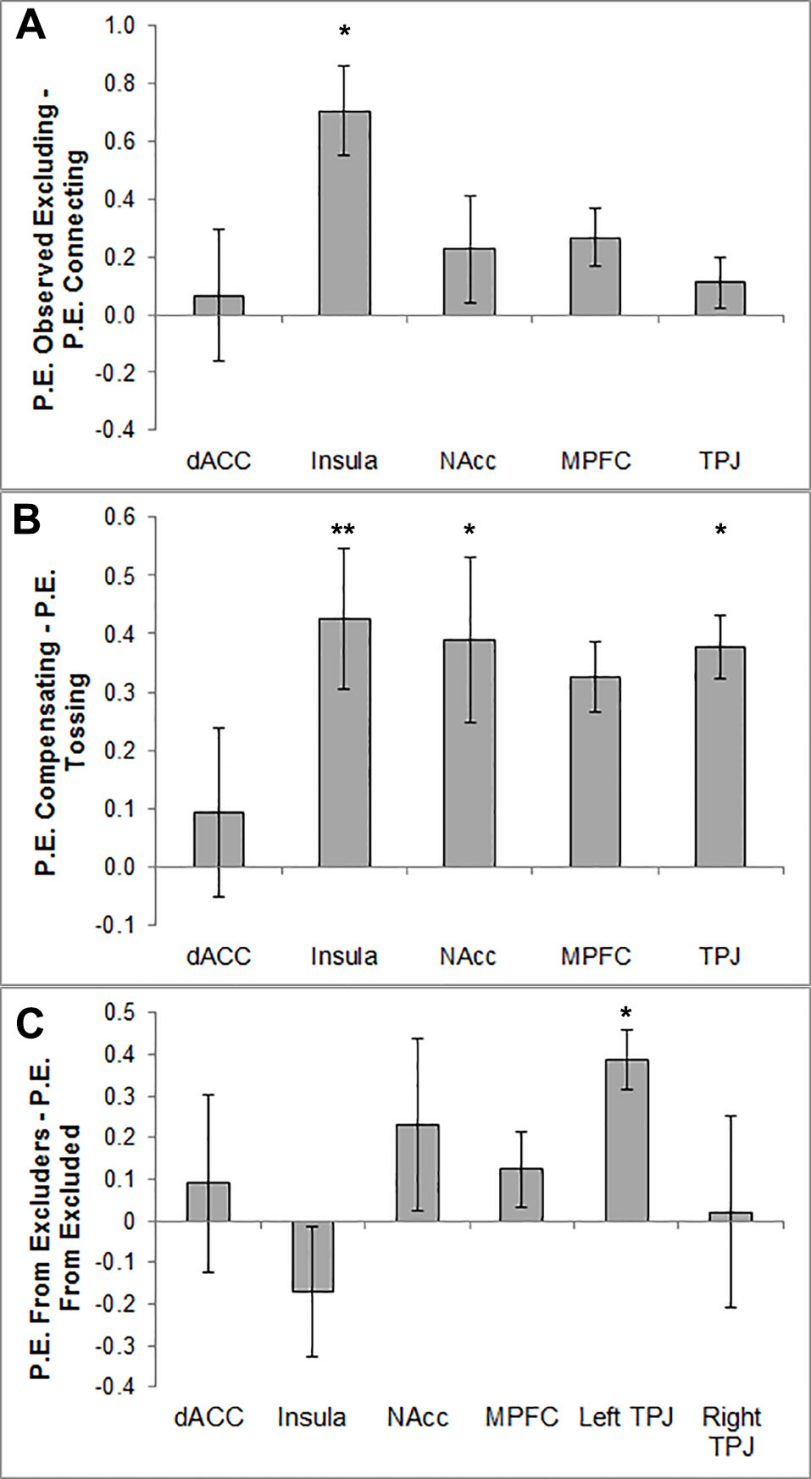
Difference scores of activity in ROIs for the three condition contrasts. P.E. = parameter estimates. One asterisk (*) indicates *p* < .05, two asterisks (**) indicate *p* < .01, and three asterisks (***) indicate *p* < .005. Error bars represent Standard Errors (SE) of the mean. In case no significant differences were found between hemispheres, findings are presented collapsed across left and right lateralized areas. (A): Difference score of activity in the ROIs for Observed Excluding–Connecting. (B): Difference score of activity in the ROIs for Compensating—Tossing. (C): Difference score of activity in the ROIs for receiving the ball From Excluders–receiving the ball From Excluded.

When participants were not tossing or receiving the ball, but were merely observing the other players, we compared Observed Excluding (players 1 and 3 toss to each other) with Connecting (player 4 tosses to player 1 or 3). We found significant differences between Observed Excluding and Connecting for left insula (*F*(1, 22) = 10.24, *p* < .005, η_p_^2^ = 0.32) and right insula (*F*(1, 22) = 10.74, *p* < .005, η_p_^2^ = 0.33), with more activation during Observed Excluding than during Connecting (see Fig 3A). No condition effects were found for dACC, bilateral TPJ, mPFC and NAcc.

#### Ball Tossing

We tested the effects of compensating by tossing to player 4 versus tossing to player 1/3.

We found a significant difference between Compensating and Tossing for left insula (*F*(1, 22) = 7.74, *p* < .05, η_p_^2^ = 0.26), right insula (*F*(1, 22) = 7.35, *p* < .05, η_p_^2^ = 0.25), left TPJ (*F*(1, 22) = 6.72, *p* < .05, η_p_^2^ = 0.23), right TPJ (*F*(1, 22) = 6.90, *p* < .05, η_p_^2^ = 0.24), left NAcc (*F*(1, 22) = 5.22, *p* < .05, η_p_^2^ = 0.19), and right NAcc (*F*(1, 22) = 8.43, *p* < .01, η_p_^2^ = 0.28) with more activation for Compensating than for Tossing (see Fig 3B). No effects were found for dACC and mPFC.

#### Ball Receiving

Finally, we tested whether there were differences between receiving the ball from excluding players 1 and 3 or from excluded player 4. For the left TPJ, we found a significant difference between receiving tosses From Excluders and From Excluded (*F*(1, 22) = 5.45, *p* < .05, η_p_^2^ = 0.199; see Fig 3C), with more activation during receiving from excluded than receiving from excluders. The other regions showed no condition effects.

#### Correlations with performance and self-reported empathy

None of the correlations between the parameter estimates for the three contexts and behavior during the Prosocial Cyberball Game were significant. In addition, none of the correlations between the parameter estimates for the three contexts and self-reported empathy were significant.

### Whole brain analysis

#### Exclusion game: Observed Exclusion

To test for the neural correlates of observed exclusion, we tested neural correlates when participants were observing the others play. The contrast “Observed Excluding > Connecting” did not result in activation at the FDR cluster-corrected threshold.

#### Exclusion game: Compensation

To test for neural correlates of prosocial behavior, we tested neural correlates when participants were tossing the ball using the contrast “Compensating > Tossing”. This contrast resulted in increased activity in a network of brain regions that are part of the social brain network, including left and right TPJ, left and right insula and NAcc (Fig 2). Other regions that were active in this contrast are presented in Table 3.

**Table 3:** Whole brain table for neural activation for the contrast Compensating in the exclusion block > Tossing in the exclusion block (cluster corrected threshold of *p* < .05 FDR-corrected, at an initial threshold of *p* < .005).

			MNI coordinates		
Name	Voxels	T-value	X	Y	Z
Left insula	136	7.63	-24	20	-11
		4.74	-15	17	-14
		4.50	-30	26	-2
Left NAcc		3.62	-6	17	-11
Right superior temporal gyrus	226	6.68	60	-16	-8
		5.48	48	-22	-2
		3.97	45	-13	-17
Left cuneus	566	5.97	-6	-97	16
Right cuneus		5.94	6	-91	-8
		5.38	12	-88	16
Right superior temporal gyrus	209	5.30	54	-52	22
Right supramarginal gyrus (assigned to IPC)		4.04	54	-37	31
Right middle temporal gyrus		3.88	60	-52	7
Left angular gyrus	275	4.94	-42	-70	46
Left supramarginal gyrus (assigned to IPC)		4.82	-54	-49	34
Left angular gyrus (assigned to IPC)		4.72	-45	-55	37
Right insula/Inferior frontal gyrus	101	4.92	42	17	-11
Right temporal lobe		3.31	51	11	-14
Right inferior frontal gyrus		2.85	42	29	-2

## Discussion

The goal of this study was to test the neural regions that are associated with prosocial behavior in a social exclusion game. Consistent with prior studies, observing exclusion was associated with increased activity in the bilateral insula [1–2, 7–8]. Thus, also in a context when the participant actively participates in the game observing exclusion leads to insula activation. When participants could compensate for exclusion, they tossed the ball more often to the excluded player than to the other players. This was associated with additional activity in the TPJ, previously associated with perspective taking [17–18] and the NAcc, previously associated with the rewarding feeling of doing good [25].

The behavioral results of this study fit with a set of prior studies that have shown that humans tend to compensate for observed distress of others [1–3]. The interpretation of this effect is that the observation of distress in others causes feelings of personal distress as well, leading to a tendency to act and decrease the observed distress. This was highlighted further in a study that used an oxytocin manipulation [4]. When participants received a dose of oxytocin before the start of the game, they showed more compensation behavior. These results support the hypothesis that individuals with more empathic feelings show more compensation behavior, as oxytocin is known for enhancing empathy [34].

The neural measures resulted in two important findings. First, compensation of excluded others was associated with elevated activity in the TPJ. The TPJ has previously been implicated in mentalizing and perspective taking [18]. Therefore, the findings suggest that compensation behavior involves thinking about what another person is thinking and feeling. The effects of TPJ were found in independently selected ROIs, and effects were further found in whole brain analyses. Notably, mPFC did not show increased activity for observed exclusion or for prosocial compensation. This effect is surprising given that prior studies showed an increase in mPFC activity during observed exclusion compared to observed inclusion [1–2]. Also, in most social reasoning studies mPFC and TPJ are concurrently activated (for a review, see [19]), and functional connectivity studies showed that these regions are functionally connected [20]. It remains unclear why mPFC was not observed in prosocial behavior in the current study. Prior studies found that this region was active during prosocial conditions [35–36]. Possibly, in the current study prosocial behavior resulted in less conflict between self- and other oriented motives, because prosocial behavior was non-costly. Future studies should unravel the role of mPFC in costly and non-costly prosocial behavior.

A second important finding was the increased activity in the NAcc for compensation of the excluded player, which was found in both the independently selected ROI analysis, and in the whole brain comparison. Several prior studies have reported that the NAcc is not only sensitive to primary rewards, such as juice or monetary rewards, but also to social rewards, such as being accepted or collaborating (for a review, see [22]). The current study suggests that compensating others may be associated with a rewarding feeling as well. In a prior study by Harbaugh, Mayr and Burghart [25] it was found that the NAcc is more active during voluntary money transfers to charity than during mandatory transfers. The current findings fit well with this study showing that the NAcc is activated when participants can choose whether they compensate for exclusion behavior or not (see also [23–24].

This study also had some limitations which should be addressed in future studies. First, the order of tasks did not allow us to test the contrast “observed exclusion > observed inclusion”, because the order of tasks was fixed (first inclusion, then exclusion). We therefore limited our analyses to the exclusion block. This may also account for the non-significant effects of dACC, given that prior studies found effects of exclusion blocks relative to inclusion blocks[11]. In future studies it will be important to present the task blocks interleaved, which would allow for a comparison of blocks of exclusion versus blocks of inclusion [37]. Second, the participants were only women. Previous studies reported that neural responses to social interactions can differ for men and women [38]. To increase power, we limited the participant selection to women. Future studies should test if the results are generalizable to men. Third, even though sufficient for testing main effects, the sample size was small for testing brain-behavior correlations. Interestingly, brain-behavior correlations were previously found in studies using similar sample sizes but other paradigms [7]. In future studies, the question should be addressed whether prosocial compensation behavior is related to self-reported empathy, and whether this relationship can also be found between neural correlates of prosocial compensation behavior and self-reported empathy. Furthermore, to fully understand the relationship between prosocial behavior and self-reported empathy, multiple informants should be used to overcome limitations of self-reports and to better determine the empathic qualities of an individual [39].

To conclude, we confirmed that hypothesis that showing compensation behavior results in increased activity in a key area of the social brain network, which is the TPJ, and in addition showed that NAcc is recruited when showing compensation behavior. In contrast, observed exclusion only resulted in insula activity, suggesting separable contributions of these brain regions. This study demonstrated eligibility of a new paradigm to test prosocial behavior which dissociated between different components of understanding why and how individuals act prosocial. These results have important implications for studies that aim at individual differences in prosocial behavior, and they may suggest a useful tool for testing effects of prosocial interventions.
